# Mandatory vaccinations in European countries, undocumented information, false news and the impact on vaccination uptake: the position of the Italian pediatric society

**DOI:** 10.1186/s13052-018-0504-y

**Published:** 2018-06-14

**Authors:** Elena Bozzola, Giulia Spina, Rocco Russo, Mauro Bozzola, Giovanni Corsello, Alberto Villani

**Affiliations:** 1 Bambino Gesù Children Hospital, Pediatric and Infectious Diseases Unit, Rome, Italy; 2 Maternity and Pediatrics Services – Local Health Units Benevento, Benevento, Italy; 3 Department of Internal Medicine and Therapeutics, University of Pavia, Pavia, Italy; 4 Mother and Child Department, University of Palermo, Palermo, Italy

**Keywords:** Vaccination, Vaccination policies, Europe, Children

## Abstract

**Background:**

High rates of vaccination coverage are important in preventing infectious diseases. Enforcing mandatory vaccinations is one of the strategies that some Countries adopted to protect the community when vaccination coverage is not satisfactory. In Italy, in 2017 vaccination against diphtheria, tetanus, pertussis, hepatitis B, poliovirus, Haemophilus influenzae type b, measles, mumps, rubella and varicella became compulsory in childhood. In order to contrast vaccination policies, anti-vaccination campaigns contribute to the spread of fake news. Among them, there is the false information that Italy is the only one country with mandatory vaccination policy. Aim of our study is confronting vaccination policies in children under 18 months against among different European countries for the following vaccines: diphtheria, tetanus, pertussis, hepatitis B, poliovirus, Haemophilus influenzae type b, measles, mumps, rubella and varicella.

**Methods:**

Information on policies of mandatory or recommended vaccinations of the European Countries were gathered by ECDC and compared to the Italian one.

**Results:**

European Countries recommend or contemplate compulsory vaccines. Among them, eleven Countries (35.4%) have mandatory vaccinations for at least one out of diphtheria, tetanus, pertussis, hepatitis B, poliovirus, Haemophilus influenzae type b, measles, mumps, rubella and varicella vaccine.

**Conclusion:**

Not only in Italy, vaccination against diphtheria, tetanus, pertussis, hepatitis B, poliovirus, Haemophilus influenzae type b, measles, mumps, rubella and varicella is mandatory in children under 18 months. Other European countries adopted compulsory policies in order to prevent the spread of infectious diseases and to protect the community.

## Background

High rates of vaccination coverage in childhood are important to prevent infectious diseases contributing to the decline in mortality and morbility. In this contest, vaccinations represent one of the most important tool of primary prevention [1].

Lack of information and fake news are actually recognized among the main factors contributing to low immunization coverage. In particular, the increasing risk of vaccine preventable disease outbreaks and the decreasing vaccine coverage could be related to vaccine hesitancy [2].

In this contest, anti-vaccination campaigns have had a damaging impact on vaccine uptake. For example, the link between Mumps-Measles-Rubella vaccine and autism described in a now-retracted article published by *The Lancet* in 1998 is still questioning the safety of vaccines [3].

Enforcing mandatory vaccinations is one of the strategies that some Countries adopted and others are considering in order to face this issue. Polices that mandate vaccinations have always been controversial and related to opposition and disputation. A recent American Survey have shown that more than 10% of parents disapproved compulsory vaccinations and was opposed to their safety and utility [4].

### Aim of our study

Aim of our study is confronting vaccination policies in children under 18 months against diphtheria, tetanus, pertussis, hepatitis B, poliovirus, Haemophilus influenzae type b, measles, mumps, rubella and varicella among different European countries.

## Methods

Information on policies of mandatory or recommended vaccinations of the 31 European Countries were gathered by ECDC and were taken from the official website (https://vaccine-schedule.ecdc.europa.eu/) and are summarized in Table 1 [5].

**Table 1:** information on policies of mandatory or recommended vaccinations of the European Countries

Country	Dhyptheria	Tetanus	Pertussis	Hepatitis B	HiB	Poliovirus	Measles	Mumbs	Rubella	Varicella
Austria	R	R	R	R	R	R	R	R	R	R
Belgium	R	R	R	R	R	Ma	R	R	R	R^a^
Bulgaria	Ma	Ma	Ma	Ma	Ma	Ma	Ma	Ma	Ma	Nr
Croatia	Ma	Ma	Ma	Ma	Ma	Ma	Ma	Ma	Ma	Nr
Cipro	R	R	R	R	R	R	R	R	R	R
Czech Republic	Ma	Ma	Ma	Ma	Ma	Ma	Ma	Ma	Ma	R
Denmark	R	R	R	R*	R	R	R	R	R	Nr
Estonia	R	R	R	R	R	R	R	R	R	Nr
Finland	R	R	R	R	R	R	R	R	R	R
France	Ma	Ma	Ma	Ma	Ma	Ma	Ma	Ma	Ma	Nr
Germany	R	R	R	R	R	R	R	R	R	R
Greece	Ma	Ma	Ma	Ma	R	R	R	R	R	R
Hungary	Ma	R*	Ma	Ma	Ma	Ma	Ma	Ma	Ma	Nr
Iceland	R	R	R	Nr	R	R	R	R	R	Nr
Ireland	R	R	R	R	R	R	R	R	R	Nr
Italy	Ma	Ma	Ma	Ma	Ma	Ma	Ma	Ma	Ma	Ma
Latvia	Ma	Ma	Ma	Ma	Ma	Ma	Ma	Ma	Ma	Ma
Lichteinstein	R	R	R	R	R	R	R	R	R	R
Lituania	R	R	R	R	R	R	R	R	R	Nr
Luxemburg	R	R	R	R	R	R	R	R	R	R
Malta	R	R	R	R	R	R	R	R	R	Nr
Netherlands	R	R	R	R	R	R	R	R	R	Nr
Norvegia	R	R	R	R	R	R	R	R	R	Nr
Poland	Ma	Ma	Ma	Ma	Ma	Ma	Ma	Ma	Ma	R^a^
Portugal	R	R	R	R	R	R	R	R	R	Nr
Romania	R	R	R	R	R	R	R	R	R	Nr
Slovakia	Ma	Ma	Ma	Ma	Ma	Ma	Ma	Ma	Ma	Nr
Slovenia	R	R	R	R	R	R	R	R	R	Nr
Spain	R	R	R	R	R	R	R	R	R	R
Sweden	R	R	R	R	R	R	R	R	R	Nr
UK	R	R	R	R	R	R	R	R	R	R^a^

*Ma* Mandatory, *R* Recommended, *Nr* Not recommended

R^a^ recommended for specific groups only

For the purpose of the study, a mandatory vaccination is defined as a vaccination that every child must receive by law without the possibility for the parent to choose to accept the uptake or not [6].

A recommended vaccination is a vaccination that is included in the national immunization program for all or for some specific groups independent of being funded or not [6].

## Results

A total of 31 European Country was analyzed.[5] In particular, a percentage of one hundred was in favor with vaccinations in childhood. Eleven Countries introduced mandatory vaccination (35.4%) and the other recommended vaccination. Latvia has ten mandatory vaccines in childhood as well as Italy. Some countries (Bulgaria, Croatia, Czech Republic, France, Hungary, Poland and Slovakia) have as many as nine vaccines which are compulsory among children. All the European Countries recommended or introduced compulsory vaccinations for the following vaccinations: tetanus, diphtheria, pertussis, Haemophilus influenza type B, Hepatitis B, poliovirus, mumps, measles, rubella with the exception of Iceland that did not recommend Hepatitis B vaccination.

### Mumps, measles, rubella

In nine Countries (Bulgaria, Croatia, Czech Republic, France, Hungary, Italy, Latvia, Poland, Slovakia) vaccination against mumps-measles-rubella is mandatory. It is recommended in the other twenty-two Countries.

### Varicella

Vaccination against varicella is mandatory in Italy and Latvia. It is recommended in eight Countries without restrictions and in three Countries only for specific groups.

### Tetanus-diphtheria-pertussis

In ten Countries (Bulgaria, Croatia, Czech Republic, France, Greece, Hungary, Italy, Latvia, Poland, Slovakia) vaccination is mandatory and is recommended in the other twenty-one Countries.

### Haemophilus influenza type B

Vaccination for Haemophilus influenza type B is mandatory in nine Countries (Bulgaria, Croatia, Czech Republic, France, Hungary Italy, Latvia, Poland, Slovakia,) and is recommended in twenty-two Countries.

### Hepatitis B

Vaccination for Hepatitis B is mandatory in nine Countries (Bulgaria, Croatia, Czech Republic, France, Hungary, Italy, Latvia, Poland, Slovakia) and it is recommended in twenty Countries. Denmark recommend vaccination just for specific groups and Iceland did not recommend it.

### Poliovirus

Poliovirus vaccination is mandatory in ten Countries (Belgium, Bulgaria, Croatia, Czech Republic, France, Hungary, Italy, Latvia, Poland, Slovakia) and recommended in all the others.

## Discussion

National and regional immunization polices are supported by vaccination coverage. In particular, it can influence the interventions done and increase or decrease vaccine uptake [7].

Considering the Italian scenario and immunization coverage data from 2010 to 2016, coverage rates have been decreasing since 2012 and are – as for now – still below the targets established by the Ministry of Health, with a great heterogeneity within the different regions [8].

With regards to Hepatitis B, tetanus, diphtheria, poliovirus, in 2016 about two third of Italian Regions had coverage rates lower than 90% [8].

In the last years, an important decreasing of vaccination coverage rates in childhood have been described for tetanus, diphtheria, pertussis, Haemophilus influenza type B, Hepatitis B, poliovirus, measles, mumps and rubella. As a consequence, due to inadequate vaccination coverage^,^ infectious diseases spread [9].

Considering, for example, measles outbreaks and European scenario, recent outbreaks have occurred in Romania (7570 cases), Italy (5006 cases), Ukraine (4667), and Germany (891 cases). A percentage of 87% of cases occurred in unvaccinated people [10].

These outbreaks have led to discussion on vaccination policies in any European Countries. All the European Union Countries have a long tradition of vaccination program but there is still much that can be done to improve and accelerate immunization coverage. In this European scenario, there are large differences between Countries considering the type of vaccine used, number of doses and timing of vaccinations but also differences in whether vaccinations are recommended or mandatory.

In eleven out of thirty-one countries there is at least one mandatory vaccine to increase immunization coverage. There are many reasons why Italy and European Countries are still not meeting immunisation needs. In particular, poor understanding and false perceptions of vaccination by the public and sometimes by health-care professionals and false informations circulating by the mass media have damaged parent’s confidence in vaccinations [11].

In Italy, until June 2017, only four vaccination were mandatory (polio, diphtheria, tetanus and hepatitis B). After a scientific among the Italian Scientific Society belonging to the “Vaccine Board” and the political community, in February 2017, the Ministry of Health issued the 2017–2019 National Immunization Prevention Plan and in July 2017 the law 119/2017 for compulsory vaccination have been approved [12–14] Italy approved a new law to enforce mandatory childhood vaccinations in 2017, so that compulsory vaccines rise to 10 [11].

As described by Burioni et al., the new law is giving good results [15]. In particular, an increase by 1 and 2.9% of polio and measles vaccine uptake respectively have been shown. Moreover, almost one-third of the previously unvaccinated children born in 2011–15 have now been immunized [16].

As for vaccination coverage, encouraging results have recently been reported: an increase of 1% from June to October 2017 for the hexavalent (tetanus, diphtheria, pertussis, Haemophilus influenza type B, Hepatitis B, poliovirus) vaccine and of 2,9% for the measles-mumps-rubella vaccine.

In order to contrast Italian vaccination policies, anti-vaccination campaigns contribute to the spread of fake news. People who had been asked to vaccinate their children have often been scared by fake news and by the false information that Italy is the only one country with mandatory vaccination policy.

“While it is preferable that high community demand and acceptance make compulsory vaccination programs unnecessary, World Health Organization (WHO) understands that some Countries may wish to move in that direction when faced with declining vaccination rates and outbreaks of disease.” Moreover, “WHO is very interested in learning from the experience of countries who introduce compulsory vaccination in order to better understand the impact on immunization coverage and the strengths and weaknesses of such approaches” [17].

## Conclusions

Not only in Italy, vaccines are mandatory in childhood. Other countries adopted compulsory policies in order to prevent the spread of infectious diseases.
